# Changes in the Plasma Apurinic/Apyrimidinic Endonuclease 1/Redox Factor-1(APE1/Ref-1) Level during Cancer Surgery: An Observational Study

**DOI:** 10.3390/medicina57111280

**Published:** 2021-11-21

**Authors:** Yumin Jo, Yeojung Kim, Eunhye Park, Yuran Lee, Jiyeon Kim, Minwoong Kang, Jaesung Lim, Insang Song, Chaeseong Lim, Byeonghwa Jeon

**Affiliations:** 1 Department of Anesthesiology and Pain Medicine, College of Medicine, Chungnam National University, Daejeon 35015, Korea; lemonny87@cnuh.co.kr (Y.J.); yeojung80@naver.com (Y.K.); beareunbear@gmail.com (E.P.); 2 Department of Physiology, College of Medicine, Chungnam National University, Daejeon 35015, Korea; lyr0913@gmail.com; 3 Department of Surgery, College of Medicine, Chungnam National University, Daejeon 35015, Korea; jykim@cnuh.co.kr (J.K.); songis@cnuh.co.kr (I.S.); 4 Department of Thoracic and Cardiovascular Surgery, College of Medicine, Chungnam National University, Daejeon 35015, Korea; dreamerkang@hanmail.net; 5 Department of Urology, College of Medicine, Chungnam National University, Daejeon 35015, Korea; uro17@cnuh.co.kr

**Keywords:** anesthesia, APEX1 protein, cancer, propofol, surgery

## Abstract

*Background and Objectives*: Propofol-based total intravenous anesthesia (TIVA) is presumed to have more favorable effects on the prognosis of patients with cancer compared with volatile inhaled anesthesia (VIA). We hypothesized that these anesthetics target plasma apurinic apyrimidinic endonuclease/redox effector factor-1 (APE1/Ref-1) as a possible mechanism of action. *Materials and Methods*: The plasma APE1/Ref-1 level was evaluated three times during surgery for cancer, i.e., before anesthesia, immediately after cancer resection, and finally, in the recovery room. Blood (3 cc) was drawn from the radial artery catheter, and plasma APE1/Ref-1 levels were compared according to measurement time and between the two groups. Spearman’s Rho correlation analysis was performed to determine relationships among body mass index, American Society of Anesthesiologists classification, age, sex, cancer type, and tumor-node-metastasis (TNM) stage. A total of 166 patients (VIA: 129; TIVA: 37) were enrolled. *Results*: Plasma APE1/Ref-1 level increased significantly (*p* = 0.028) after cancer resection compared with before surgery, but no significant difference was observed between anesthetics (*p* = 0.134). The post-resection plasma APE1/Ref-1 level showed a positive correlation with the NM stages, but not the T stage. *Conclusions*: The plasma APE1/Ref-1 level increased during surgery with more severe lymph node invasion, but there were no significant differences according to the anesthetics used.

## 1. Introduction

For patients with cancer, propofol-based total intravenous anesthesia (TIVA) helps to preserve immunity more effectively than volatile inhaled anesthesia (VIA) [1,2,3]. Evidence of propofol as an advantageous drug for long-term prognosis has also been found [4]. Propofol was reported to suppress cell invasion by decreasing hypoxia-inducible factor 1α (HIF-1α) expression in LPS-treated non-small cell lung cancer cells [3]. Many in vitro studies have shown that the underlying mechanism involves directly targeting the RNA of cancer cells [5]; however, there have been few in vivo studies, and the results of human studies are inconsistent [6,7]. Therefore, we examined the mechanism by which anesthetics affect the outcomes of cancer surgery.

Apurinic/apyrimidinic endonuclease 1 (APE1), also designated as redox effector factor-1 (Ref-1), is a 37-kDa nuclear protein involved in the base excision repair pathway of damaged bases and DNA single-strand breaks caused by endogenous and exogenous oxidative stress. APE1/Ref-1 acts as a reductive activator of many transcription factors involved in apoptosis, inflammation, angiogenesis, and survival pathways [8,9,10,11], and has attracted attention as a biomarker for the diagnosis of various cancers, because it is expressed in malignant tumors and increases in plasma with increasing cancer stage [12,13,14]. In addition, APE1/Ref-1 in the plasma appears to be involved in anti-inflammatory activity [15]. HIF-1 is involved in the mechanism of action of propofol, and APE1/Ref-1 interact with each other [16,17]. Therefore, we postulated that the plasma APE1/Ref-1 level would differ according to the anesthetic agent used in cancer surgery. Here, we investigated whether changes in the plasma APE1/Ref-1 level reflect another mechanism of action by which anesthetics influence cancer prognosis. We hypothesized that TIVA would reduce the plasma APE1/Ref-1 level during cancer surgery compared with VIA. In addition, we examined the changes in plasma APE1/Ref-1 level over time and other factors that affect its level during cancer surgery.

## 2. Materials and Methods

### 2.1. Study Population

This study was approved by the institutional review board of Chungnam National University Hospital (Approval No. CNUH 2017-05-027; Daejeon, Korea) and is registered with the Clinical Research Information Service (cris.nih.go.kr, Trial Registration No. KCT0002650). This study was conducted in a single tertiary hospital, and written informed consent to participate was obtained from all patients. It was initially designed as a randomized controlled study, but many anesthesiologists preferred inhalation anesthesia; therefore, we could not proceed with the original protocol and switched to an observational study method. There are still many anesthesiologists who prefer gas anesthesia, because TIVA requires more attention to the maintenance of the veinous line, and the recovery of consciousness is slower.

The patient criteria were as follows: diagnosis with colon, liver, lung, kidney, or prostate cancer before surgery; planned regular surgery for cancer resection; and expectation of an arterial catheter due to patient monitoring during surgery. Patients were excluded based on the following criteria: age less than 18 years and reoperation. Conditions excluded from the enzyme-linked immunoassay (ELISA) comparison were those with postoperative histopathological confirmation as benign and those with a gap of more than 1 year between the time of blood sampling and the time of ELISA analysis. On the day before surgery, an anesthesiologist provided an anesthesia consent form to each patient and explained the study. The study start date for the first patient was 1 July 2017, and the last patient’s blood was stored on July 31, 2018. 

### 2.2. Anesthesia

All patients were administered an intramuscular injection of 0.2 mg glycopyrrolate at 30 min before surgery. Patients under 65 years of age were also administered an intramuscular injection of 2 mg midazolam. In patients older than 65 years of age, the midazolam dose was reduced to 1 mg. Patients who signed the study consent form were anesthetized in the manner preferred by their anesthesiologist. Maintenance of anesthesia during cancer surgery was performed by one of two methods. Anesthesia was induced after the establishment of routine noninvasive monitoring with the bispectral index monitor. Invasive arterial blood pressure monitoring was performed as planned. For the TIVA group, an initial target concentration of 4.0 μg/mL propofol (effect-site targeting, Schnider model) was administered intravenously using a target-controlled infusion device (Orchestra Base Primea; Fresenius Vial, Brézins, France). For the VIA group, 1–2 mg/kg propofol was administered intravenously to induce anesthesia. After the loss of consciousness, adequate mask ventilation was confirmed; 0.8 mg/kg rocuronium was administered intravenously for neuromuscular blockade under the guidance of peripheral neuromuscular transmission monitoring. A fixed target concentration of 4.0 ng/mL remifentanil (plasma site, Minto model for target-controlled infusion) was administered intravenously and maintained until the end of surgery. Tracheal intubation was performed at a train-of-four count of zero. Additional rocuronium was administered with neuromuscular monitoring guidance. Anesthesia was maintained with propofol using target-controlled infusion for the TIVA group, and with sevoflurane or desflurane inhalation for the VIA group. The main anesthetics were controlled to maintain the bispectral index between 40 and 60.

The main anesthetic agents differed according to the anesthesiologist’s preference, but the anesthetic protocol was otherwise identical. At the start of subcutaneous suture, intravenous patient-controlled analgesia (PCA) began. The total PCA volume was 100 mL, consisting of 1500 μg (30 mL) fentanyl, 0.6 mg (4 mL) ramosetron, and 66 mL normal saline. The PCA device (Gemstar Pump; Hospira, Lake Forest, IL, USA) was programmed to deliver 1 mL as the basal infusion rate and 1 mL on demand; the lock-out time was 15 min. PCA rounding is performed daily at 3 PM to check for side effects and to adjust the dosage. A bolus of 0.3 mg ramosetron was intravenously administered. At the end of surgery, the administration of propofol or gas with remifentanil was stopped for all patients. Neuromuscular block was antagonized with an optimal dose of sugammadex based on the train-of-four monitoring. After tracheal extubation, patients were moved to the recovery room. Arterial blood gas analysis was performed to determine whether the exit criteria were met in the recovery room and whether oxygen therapy was needed in the ward. At this time, a third sample was performed with ABGA. 

### 2.3. Blinded Analysis

The surgeon was blinded to the drugs used to maintain the anesthesia. In addition, the researchers who collected the blood samples and those who later performed ELISA analysis were blinded to the anesthetic agent used.

### 2.4. Blood Samples

Arterial blood samples were collected three times (before anesthesia, immediately after cancer resection, and finally, in the recovery room) only in patients with arterial catheter insertion for surgery and anesthesia. Blood (3 cc) was drawn from the radial artery catheter and collected into heparin-coated tubes. Plasma was separated by centrifugation at 3,000 rpm for 10 min at room temperature, and then re-centrifuged at 5000 rpm for 5 min to obtain cell-free plasma, which was stored in liquid nitrogen until use. 

### 2.5. Plasma APE1/Ref-1 Measurements

Plasma APE1/Ref-1 levels were measured using the Human APE1/Ref-1 ELISA kit (MediRedox Inc., Daejeon, Korea). All procedures were performed in accordance with the manufacturer’s instructions. All samples were tested twice. The average of the measured values was calculated and compared.

### 2.6. Statistical Analyses

#### 2.6.1. Sample Size

Using G*power software, the target numbers of patients were calculated to be 35 (TIVA) versus 105 (VIA) using the Mann–Whitney test, effect size d = 0.5, α error prob = 0.05, power = 0.8, and allocation ratio = 3. When these numbers of patients had been enrolled, the study was terminated. The allocation ratio was established based on the TIVA:VIA ratio of approximately 1:3 for the past 3 years in our hospital.

#### 2.6.2. Comparison between Two Groups

Independent two-tailed Student’s t-tests were used to compare means of continuous, normally distributed data between the TIVA and VIA groups. The chi-squared test was used to compare means of categorical variables (e.g., sex and cancer type) between the two groups. Continuous data are presented as means ± standard deviation (SD). For categorical variables, the numbers of patients (n) and proportions (%) were calculated. SPSS Statistics, version 24.0 (SPSS Inc., Chicago, IL, USA) was used to evaluate the data.

#### 2.6.3. Propensity Matching

To adjust for possible selection bias (e.g., cancer type) and confounding factors, 1:1 ratio propensity score matching was performed using the MatchIt package in R. Serum APE1 level can differ among cancers, and the numbers of anesthetic methods also differed among cancers. Therefore, we matched each type of cancer using nearest neighbor matching, which matches the absolute differences of estimated propensity scores of all patients in both groups from the smallest to largest difference. The absolute standardized difference was calculated to validate the suitability of balanced propensity score matching diagnostics between the two groups; an absolute standardized difference <0.1 for the covariate indicated that the two groups were sufficiently balanced. Categorical data were compared using the chi-squared test or Fisher’s exact test as appropriate, with the results expressed as numbers (%).

#### 2.6.4. Comparison by Measurement Time within the Group

Each paired *t*-test of data from the 166 patients without propensity matching was performed to analyze changes over time.

#### 2.6.5. Correlation Analysis

Spearman’s Rho correlation analysis of data from the 166 patients was performed to identify correlations of plasma APE1/Ref-1 level with body mass index (BMI), American Society of Anesthesiologists (ASA) class, age, sex, cancer type, and tumor-node-metastasis (TNM) stage. For the TNM stage, information regarding the pathology recording sheet confirmed after surgery was used, and statistical analyses were performed by dividing the stages into numbers as shown in Table 1.

#### 2.6.6. Comparison by Type of Cancer

Repeated measures analysis of variance was performed regarding the data of the 166 patients prior to propensity matching to analyze whether changes over time were significantly different depending on the type of cancer.

#### 2.6.7. Non-Parametric Analysis

As plasma APE1/Ref-1 levels did not follow a normal distribution, a non-parametric test was performed using R to ensure statistical accuracy. To evaluate the intraoperative changes in plasma APE1/Ref-1 level over time, a linear mixed effects model was used with time as the fixed effect and individuals as the random effect. To determine the effects of anesthetics (TIVA or VIA) and cancer type (colon, liver, lung, prostate, and renal cancer) on the intraoperative change in plasma APE1/Ref-1 level, two mixed models were assessed using the abovementioned variables (anesthetics and diagnosis), time, and their interactions as fixed effects. Differences were considered statistically significant if the null hypothesis could be rejected with >95% confidence interval (*p* < 0.05).

## 3. Results

**Figure 1:**
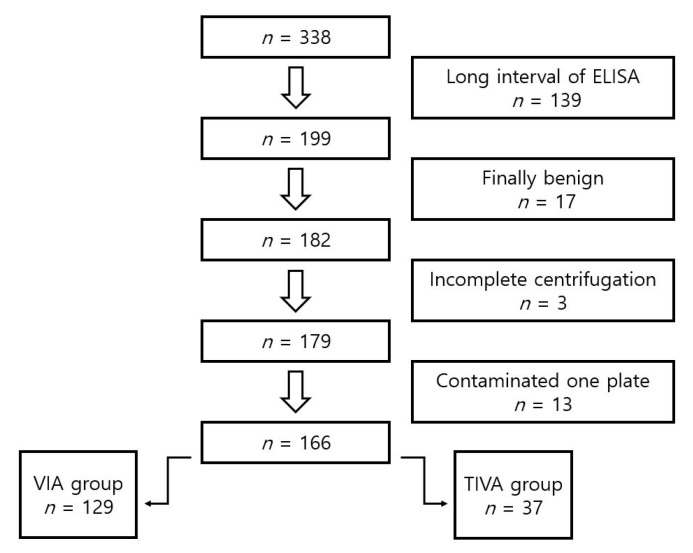
Patient flow chart.

The flow chart is shown in Figure 1. After the exclusion of ineligible patient samples, gas anesthesia was performed in 129 patients, and TIVA was performed in 37 patients. Demographic data are shown in Table 2. The patient sex distribution was more than 70% men and did not differ between the two groups. The total intraoperative remifentanil dose was similar between VIA (2.2 ± 0.7 mg) and TIVA (2.1 ± 0.7 mg). There was a significant difference between the two groups regarding cancer type (*p* = 0.043), and propensity score matching was necessary for the comparison between two groups. There was no significant difference between surgery time and anesthesia time after propensity matching (Table 2).

### 3.1. Plasma APE1/Ref-1 Levels in The Overall Cohort

**Figure 2:**
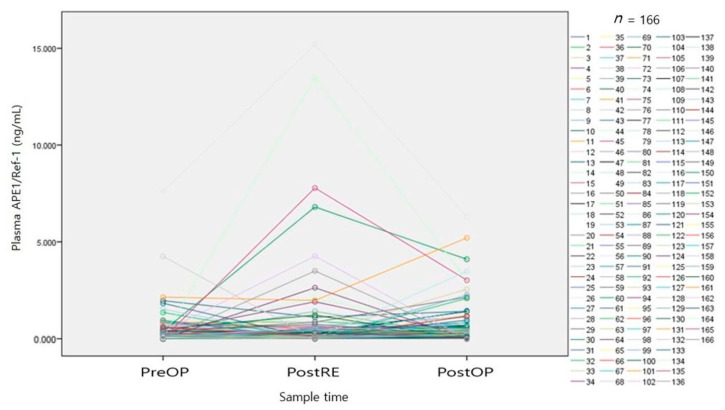
Test values of overall cohort (166 patients) in spaghetti plot format. PreOP, preoperative sample; PostRE, postresection sample; PostOP, postoperative sample.

Figure 2 shows the three test values of 166 patients in a spaghetti plot format. Most plasma APE1/Ref-1 values were less than 2.0 ng/mL; test values immediately after removal of the mass (PostRE) tended to increase, compared with the preoperative values.

### 3.2. Comparison of Plasma APE1/Ref-1 Levels between Two Groups

**Figure 3:**
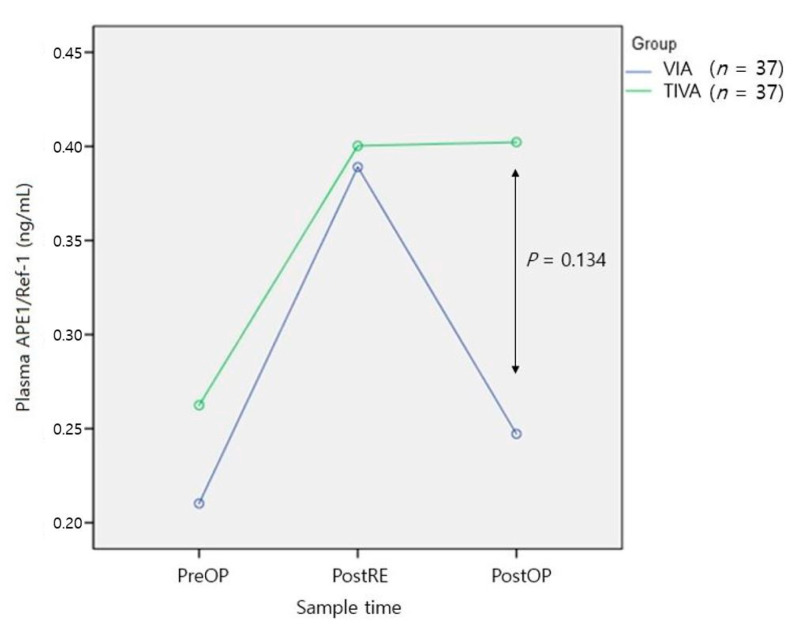
Comparison of plasma APE1/Ref-1 levels between two groups. VIA, volatile inhaled anesthesia; TIVA, total intravenous anesthesia; PreOP, preoperative sample; PostRE, postresection sample; PostOP, postoperative sample.

Figure 3 shows the results of plasma APE1/Ref-1 analysis in 37 patients per group after propensity matching involving multiple variables, including cancer type. The preoperative means ± SD were similar between groups (0.21 ± 0.03 in the VIA group and 0.26 ± 0.04 in the TIVA group; *p* = 0.40). Immediately after resection, the values tended to increase in both groups (0.38 ± 0.12 in the VIA group and 0.40 ± 0.10 in the TIVA group) but did not differ significantly between the two groups (*p* = 0.94). Immediately after surgery, the value in the VIA group decreased to 0.24 ± 0.05 but was 0.40 ± 0.08 in the TIVA group, similar to the value immediately after resection; these values did not differ significantly between the two groups (*p* = 0.134).

### 3.3. Comparison of Plasma APE1/Ref-1 Level according to Measurement Time 

**Figure 4:**
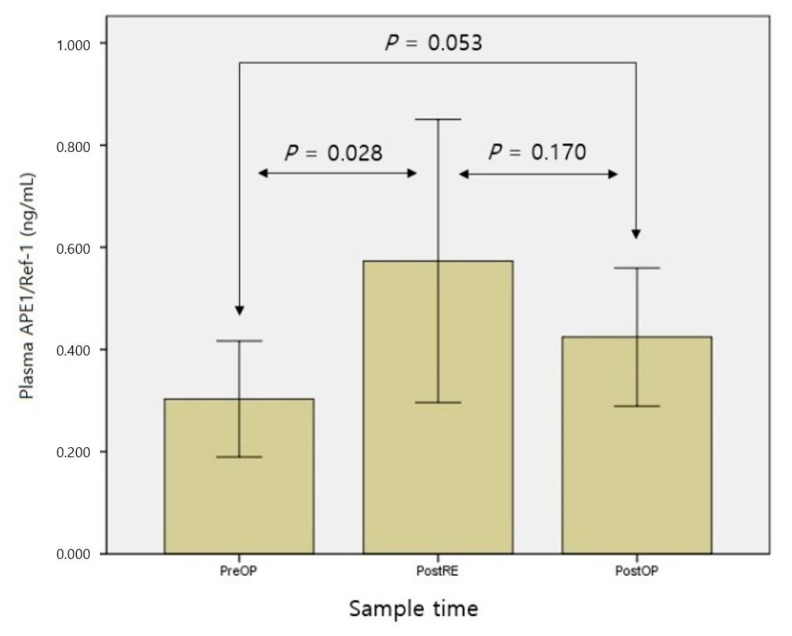
Comparison of plasma APE1/Ref-1 level according to measurement time. PreOP, preoperative sample; PostRE, postresection sample; PostOP, postoperative sample.

The means ± SD of plasma APE1/Ref-1 levels were 0.30 ± 0.05 preoperatively, 0.57 ± 0.14 after resection, and 0.42 ± 0.06 after surgery. As shown in Figure 4, paired t-tests were performed to analyze changes over time. Plasma APE1/Ref-1 level increased significantly after cancer resection compared with before surgery (*p* = 0.028). The level tended to decrease again after surgery, but this change was not statistically significant.

### 3.4. Analyses of Variables Correlated with Plasma APE1/Ref-1 Level

**Figure 5:**
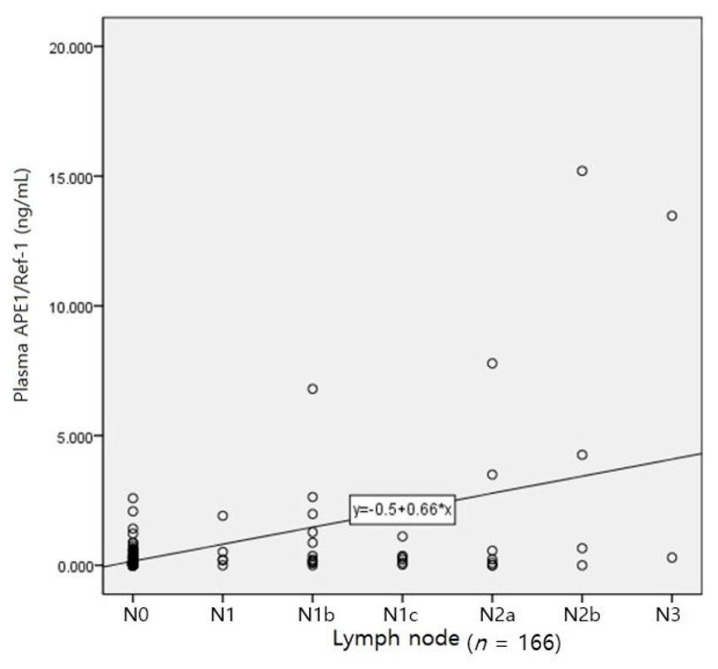
Correlation of lymph node invasion grade and intraoperative plasma APE1/Ref-1 level (immediately after cancer resection).

The relationships of plasma APE1/Ref-1 with BMI, ASA class, age, sex, T, N, and M are shown in Table 3. Female patients had higher BMI (*r* = 0.166, *p* = 0.032), and older female patients had a higher ASA class (*r* = 0.220, *p* = 0.004). Female patients were older (*r* = 0.220, *p* = 0.004), and the extent of lymph node involvement was severe (*r* = 0.192, *p* = 0.013). Plasma APE1/Ref-1 levels before surgery and after cancer resection increased with increasing severity of lymph node involvement. In particular, after lesion resection, a positive correlation was observed (*r* = 0.300, *p* < 0.001), but this correlation disappeared after surgery. Regarding metastasis to other organs, positive correlations were evident after resection (*r* = 0.243, *p* = 0.002) and after surgery (*r* = 0.154, *p* = 0.048). Figure 5 shows the relationship between plasma APE1/Ref-1 level after resection and the degree of lymph node invasion, which showed the highest correlation (*r*^2^ = 0.255).

### 3.5. Change in Pattern of Plasma APE1/Ref-1 according to Cancer Type

**Figure 6:**
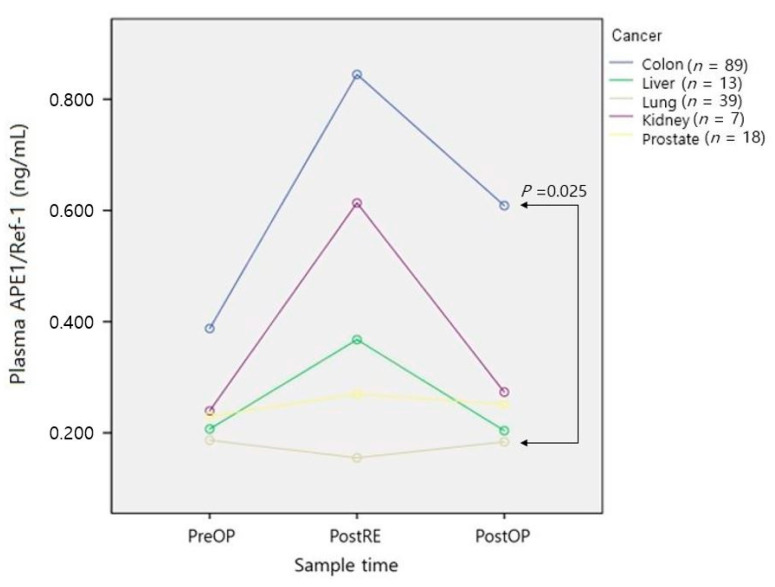
Changes in plasma APE1/Ref-1 level according to cancer type. PreOP, preoperative sample; PostRE, postresection sample; PostOP, postoperative sample.

As shown in Figure 6, patients with colon cancer showed higher APE1/Ref-1 levels from the preoperative stage, compared with patients who had other cancers. Patients with lung cancer tended to have decreased plasma APE1/Ref-1 levels after resection of the cancer lesion. There were minimal changes over time in patients with prostate cancer. The only significant difference was observed between patients with colon cancer and patients with lung cancer (*p* = 0.025).

### 3.6. Non-Parametric Analysis

Table 4 shows the results of analyses of the interaction model according to the sample time and anesthetic method (VIA and TIVA). The change with time was significant (*p* = 0.005), but there was no significant difference according to the type of anesthesia (*p* = 0.832), interaction (*p* = 0.199), or cancer type (*p* = 0.63). Only the preoperative plasma APE1/Ref-1 level showed a significant difference (*p* = 0.049) according to cancer type, but this difference disappeared during surgery. This was the only difference from the results of parametric analysis (Figure 6), and in all other cases, parametric analysis and nonparametric analysis showed the same significance. Figures and results that are not included in the text are provided in the Supplementary Materials.

## 4. Discussion

Plasma APE1/Ref-1 level increased during surgery but did not differ significantly between the two groups. Plasma APE1/Ref-1 level showed a positive correlation with the degree of lymph node involvement. Among the five types of cancer, patients with colon cancer had the highest plasma APE1/Ref-1 level, while patients with lung cancer had the lowest level. These findings should be interpreted carefully, because this study only included patients with cancer who had an indication for surgery, rather than all patients diagnosed with cancer.

APE1/Ref-1 is an intracellular protein that repairs DNA damage and regulates transcription factor activity. It mainly exists in the nucleus and translocate to the cytoplasm after acetylation within the cell; it can also be released into the plasma. Increased APE1/Ref-1 expression in tissue is correlated with the degree of cancer progression and with a decrease in radiosensitivity [18]. A study using small interfering RNA (siRNA) indicated that a reduced level of APE1/Ref-1 markedly delayed the growth of ovarian cancer both in vitro and in vivo [19]. 

In correlation analysis (Table 3), plasma APE1/Ref-1 levels before surgery and after cancer resection increased with increasing severity of lymph node involvement. In particular, after lesion resection, a positive correlation was observed (*r* = 0.300, *p* < 0.001). Although no patients with gastric cancer were included in our study, plasma APE1/Ref-1 has been identified as a valuable marker for the prediction of lymph node metastases in patients with gastric cancer [20]. Plasma APE1/Ref-1 levels were increased in blood samples collected immediately after lesion removal and were strongly correlated with the degree of lymph node invasion; therefore, the surgical identification, exfoliation, and resection of the lesion are presumably the cause of increased plasma APE1/Ref-1.

Comparisons between the two groups performed via propensity matching did not show significant differences between the two groups. However, Figure 3 shows that the TIVA group tended to maintain the increased level during surgery, and the VIA group tended to show an immediate decrease in the level of plasma APE1/Ref-1 after surgery. These findings did not support our hypothesis that plasma APE1/Ref-1 would be lower in the TIVA group than in the VIA group during surgery. Moreover, the findings did not support our expectation that the anti-inflammatory and antioxidative effects of propofol would inhibit the increase in plasma APE1/Ref-1. There was no difference in plasma APE1/Ref-1 level between groups during surgery; notably, there was a difference between groups after surgery, but it was not statistically significant. As the main anesthetics were sevoflurane, desflurane, and propofol, all of which demonstrate rapid reversal [21], the residual effect of the anesthetic agent cannot explain this phenomenon.

In our study, patients with colorectal cancer had the highest plasma APE1/Ref-1 level, followed by patients with kidney cancer, prostate cancer, liver cancer, and lung cancer. In 2018, Huajun reported that patients with colon cancer had significantly higher plasma anti-APE1/Ref-1 autoantibody levels (2.68 ± 0.34 ng/mL) compared with the control group (1.83 ± 0.31 ng/mL; *p* < 0.001) [22]. In 2019, Kim suggested using a plasma APE1/Ref-1 level > 0.202 ng/mL (sensitivity: 82.5; specificity: 97.4) as a diagnostic criterion to differentiate patients with clear cell renal cell cancer from healthy people [23]. In a 2019 report [12], the plasma APE1/Ref-1 expression level was significantly higher in patients with hepatocellular carcinoma (75.8 pg/mL) than in patients with liver cirrhosis (29.8 pg/mL) or controls (10.8 pg/mL) (*p* < 0.001). Although increased expression of APE1/Ref-1 by nucleus or cytoplasmic staining has been reported in patients with prostate cancer [24,25], no studies have been published regarding changes in plasma APE1/Ref1 level in patients with prostate cancer. Zhang [26] reported a significant elevation of plasma APE1/Ref-1 in patients with non-small-cell lung carcinoma, compared with controls (median: 0.159 vs. 0.091 ng/mL, respectively; *p* < 0.001). However, the previous studies used different ELISA methods or kits, as well as different measurement units; the results are therefore provided in Table 5 for a more detailed comparison. APE1/Ref-1 is a cancer-related gene, but despite FDA approval as a drug target, it shows low tissue and cell type specificity. The plasma level of APE1/Ref-1 was increased immediately after removal of the tumor, and there appeared to be no difference according to the anesthesia method used in cancer surgery.

This study had several limitations. First, the number of patients included was small, as we only included patients with arterial catheterization. As it was not a randomized controlled trial (RCT), the difference in number of patients between the two groups was also large. University hospitals still show a preference for VIA over TIVA, and there may have been bias in the comparisons between the two groups, although propensity matching was performed to resolve this issue. There was no bias in intragroup comparisons or other analyses. In all figures, values are expressed as means, but median values would have been preferable, because plasma APE1/Ref-1 level does not show a normal distribution. However, in terms of statistical significance, both parametric and nonparametric analyses showed the same results. Figures and results of nonparametric analyses not included in the text are provided in the Supplementary Materials. The final problem was the low specificity of APE1/Ref-1. We think that studying only one cancer is a way to compensate for the low specificity. Additionally, if the in vitro results on the effects of anesthetics on APE1/Ref-1 had been established, the analysis of the results would have been simple. 

## 5. Conclusions

The plasma APE1/Ref-1 level increased significantly during cancer surgery compared to before surgery. However, it did not differ significantly between patients receiving TIVA and VIA. The plasma APE1/Ref-1 level was positively correlated with the degree of lymph node invasion. Although further research is needed, the results of this study suggest that anesthetics may not affect plasma APE1/Ref-1 level during surgery in patients with cancer.

## Figures and Tables

**Table 1:** Conversion of TNM stages to numerical scores for statistical analysis.

Score	T	N	M
1	Tis	N0	M0
2	T1	N1	M1a, b
3	T1b	N1b	M1c
4	T1c	N1c	
5	T2	N2a	
6	T2b	N2b	
7	T2c	N3	
8	T3		
9	T4a		

TNM stages were converted to numerical scores for Spearman’s Rho correlation analysis of factors associated with plasma APE1/Ref-1 level.

**Table 2:** Patient demographic data and anesthesia information.

	Overall Patients			Matched Patients		
Variables	VIA (*n* = 129)	TIVA (*n* = 37)	*p*-Value	VIA (*n* = 37)	TIVA (*n* = 37)	*p*-Value
	Mean (SD)	Mean (SD)		Mean (SD)	Mean (SD)	
Age (years)						
	66 (8.7)	66 (8.9)	0.771	68 (5.3)	67 (8.9)	0.266
Height (cm)						
	162 (8.3)	161 (7.4)	0.455	160 (7.9)	161 (7.4)	0.561
Weight (kg)						
	63 (10.1)	64 (11.8)	0.771	65 (10.9)	64 (11.8)	0.615
BMI (kg/m^2^)						
	24 (3.4)	24 (3.3)	0.491	25 (3.3)	24 (3.3)	0.258
Anesthesia						
OP time (min)	199 (85.1)	170 (55.8)	0.015 *	192 (83.0)	170 (55.8)	0.194
AN time (min)	235 (88.0)	208 (60.8)	0.032 *	226 (87.3)	208 (60.8)	0.293
Propofol (mg)		1424 (428)			1424 (428)	
Remifentanil (mg)	2.2 (0.7)	2.1 (0.7)	0.592	2.1 (0.7)	2.1 (0.7)	0.870
	*n* (%)	*n* (%)		*n* (%)	*n* (%)	
Sex						
Male Female	93 (72) 36 (28)	28 (75) 9 (25)	0.668	26 (70) 11 (30)	28 (75) 9 (25)	0.794
ASA						
I II III IV	7 (5) 101 (78) 19 (15) 2 (2)	1 (3) 31 (83) 5 (14) 0	0.859	1 (3) 25 (67) 11 (30)	1 (3) 31 (83) 5 (14)	0.235
Cancer						
Colon Liver Lung Kidney Prostate	73 (57) 12 (9) 28 (22) 4 (3) 12 (9)	16 (43) 1 (3) 11 (30) 3 (8) 6 (16)	0.043 *	18 (49) 4 (11) 7 (19) 2 (5) 6 (16)	16 (43) 1 (3) 11 (30) 3 (8) 6 (16)	0.557

ASA, American Society of Anesthesiologists; BMI, body mass index; VIA, volatile inhaled anesthesia; TIVA, total intravenous anesthesia; OP, operation; AN, anesthesia. * *p* < 0.05.

**Table 3:** Correlation analysis of overall patients (Spearman’s Rho).

		BMI	ASA	Age	Sex	T	N	M
BMI (kg/m^2^)	*r*	1.000	0.083	0.020	0.166 *	−0.107	−0.049	−0.121
	*p*		0.289	0.794	0.032	0.170	0.528	0.122
ASA (I/II/III/IV)	*r*	0.083	1.000	0.220 **	0.004	0.034	0.031	0.042
	*p*	0.289		0.004	0.955	0.666	0.693	0.591
Age (years)	*r*	0.020	0.220 **	1.000	0.210 **	0.036	0.072	0.041
	*p*	0.794	0.004		0.007	0.641	0.355	0.597
Sex (M:1, F:2)	*r*	0.166 *	0.004	0.210 **	1.000	0.006	0.192 *	−0.055
	*p*	0.032	0.955	0.007		0.941	0.013	0.483
T	*r*	−0.107	0.034	0.036	0.006	1.000	0.367 **	0.281 **
	*p*	0.170	0.666	0.641	0.941		0.000	0.000
N	*r*	−0.049	0.031	0.072	0.192 *	0.367 **	1.000	0.366 **
	*p*	0.528	0.693	0.355	0.013	0.000		0.000
M	*r*	−0.121	0.042	0.041	−0.055	0.281 **	0.366 **	1.000
	*p*	0.122	0.591	0.597	0.483	0.000	0.000	
PreOP	*r*	−0.016	−0.009	0.008	−0.074	0.079	0.165 *	0.135
	*p*	0.839	0.907	0.915	0.342	0.315	0.033	0.083
PostRE	*r*	−0.025	0.051	−0.104	−0.021	0.083	0.300 **	0.243 **
	*p*	0.745	0.515	0.181	0.789	0.287	0.000	0.002
PostOP	*r*	−0.062	0.088	−0.015	−0.169 *	0.089	0.084	0.154 *
	*p*	0.425	0.257	0.852	0.030	0.252	0.282	0.048

Data are *r* (*p* value). ASA = American Society of Anesthesiologists; BMI = body mass index; PreOP = preoperative plasma APE1/Ref-1; PostRE = postresection plasma APE1/Ref-1; PostOP = postoperative plasma APE1/Ref-1. * *p* < 0.05; ** *p* < 0.01.

**Table 4:** Interaction model of sample time and anesthesia type.

	Sum Square	Mean Square	NumDF	*F* Value	*p*
Time	8.78	4.39	2	5.23	0.005 **
Anesthesia type	0.03	0.03	1	0.04	0.832
Time × Anesthetic type	2.71	1.35	2	1.61	0.199

** *p* < 0.01.

**Table 5:** Plasma APE1/Ref-1 levels in other studies.

Cancer	Author	Control level	Plasma APE1/Ref-1	Our Study (ng/mL)
Colon	Huajun	1.83 ± 0.31 ng/mL	2.68 ± 0.34 ng/mL	0.38 ± 0.10
Kidney	Kim	<0.202 ng/mL	>0.202 ng/mL	0.24 ± 0.07
Prostate				0.23 ± 0.05
Liver	Pascut	10.8 pg/mL	75.8 pg/mL	0.20 ± 0.07
Lung	Zhang	0.091 ng/mL	0.159 ng/mL	0.18 ± 0.04

Values of our study are mean ± SE of preoperative sample. In the case of prostate cancer, there were studies in tissues, but no studies in serum or plasma were found.

## Data Availability

The data presented in this study are available on request from the corresponding author.

## Supplementary Materials

The following are available online at https://www.mdpi.com/article/10.3390/medicina57111280/s1.
