# Supplementary material for: Changes in the Plasma Apurinic/Apyrimidinic Endonuclease 1/Redox Factor-1(APE1/Ref-1) Level during Cancer Surgery: An Observational Study
Source: Medicina (Kaunas). 2021 Nov 21;57(11):1280. doi: 10.3390/medicina57111280 (PMC8623191; doi:10.3390/medicina57111280)
Supplement: Supplementary file 1 [file medicina-57-01280-s001.zip › supplementary files/result_1113 (2).html]

redox


# redox

#### ocha

#### 2020 11 13

#package

```
## 
## Attaching package: 'dplyr'
```

```
## The following objects are masked from 'package:stats':
## 
##     filter, lag
```

```
## The following objects are masked from 'package:base':
## 
##     intersect, setdiff, setequal, union
```

```
## Loading required package: lme4
```

```
## Loading required package: Matrix
```

```
## 
## Attaching package: 'Matrix'
```

```
## The following objects are masked from 'package:tidyr':
## 
##     expand, pack, unpack
```

```
## 
## Attaching package: 'lmerTest'
```

```
## The following object is masked from 'package:lme4':
## 
##     lmer
```

```
## The following object is masked from 'package:stats':
## 
##     step
```

```
## Registered S3 methods overwritten by 'car':
##   method                          from
##   influence.merMod                lme4
##   cooks.distance.influence.merMod lme4
##   dfbeta.influence.merMod         lme4
##   dfbetas.influence.merMod        lme4
```

```
## Registered S3 method overwritten by 'GGally':
##   method from   
##   +.gg   ggplot2
```

```
## sROC 0.1-2 loaded
```

```
## 
## Attaching package: 'scales'
```

```
## The following object is masked from 'package:moonBook':
## 
##     comma
```

#data

```
dd<-read.csv("test_1101.csv",header=T)
colnames(dd)<-tolower(colnames(dd))

##class
str(dd)
```

```
## 'data.frame':    166 obs. of  18 variables:
##  $ patient_number: int  1692331 1687466 1687061 1687033 1681459 1679547 1676538 1675509 1675030 1673781 ...
##  $ age           : int  71 78 68 61 48 65 68 62 75 53 ...
##  $ sex           : int  1 2 1 2 1 1 1 2 1 1 ...
##  $ ht            : num  158 151 161 161 171 ...
##  $ wt            : num  65 65 70 80 72.4 ...
##  $ bmi           : num  26.2 28.4 27 30.9 24.9 ...
##  $ asa           : int  2 2 2 2 1 2 2 2 2 2 ...
##  $ diagnosis     : int  3 3 3 3 4 3 1 2 2 5 ...
##  $ pre_chemo     : int  0 0 0 0 0 0 0 0 0 0 ...
##  $ pre_rt        : int  0 0 0 0 0 0 0 0 0 0 ...
##  $ anesthetics   : chr  "d" "d" "d" "s" ...
##  $ pathology     : chr  "Adenocarcinoma" "Adenocarcinoma" "Adenocarcinoma" "Adenocarcinoma" ...
##  $ t             : int  2 8 8 7 8 5 7 7 7 8 ...
##  $ n             : int  1 1 1 1 1 1 1 1 1 1 ...
##  $ m             : int  1 1 1 1 1 1 1 1 1 1 ...
##  $ var_1         : num  0 0 0 0 0 0 0 0 0 0.03 ...
##  $ var_2         : num  0.096 0 0 0.055 0 0 0 0.047 0 0 ...
##  $ var_3         : num  0 0.033 1.169 0 0 ...
```

```
dd$patient_number<-as.factor(dd$patient_number)
dd$sex<-as.factor(ifelse(dd$sex==1,"m","f"))
dd$asahigh<-as.factor(ifelse(dd$asa>2,1,0))
dd$asa<-as.factor(dd$asa)
dd$diagnosis<-as.factor(ifelse(dd$diagnosis==1,"colon",
                               ifelse(dd$diagnosis==2,"liver",
                                      ifelse(dd$diagnosis==3,"lung",
                                             ifelse(dd$diagnosis==4,"renal","prostate")))))
dd$diagnosis<-as.factor(dd$diagnosis)
dd$pre_chemo<-as.factor(dd$pre_chemo)
dd$pre_rt<-as.factor(dd$pre_rt)
dd$anesthetics<-as.factor(ifelse(dd$anesthetics=="d"|dd$anesthetics=="s","inhalation","tiva"))
dd$pathology<-as.factor(dd$pathology)


names(dd)[13]<-paste("t_stage")
names(dd)[14]<-paste("n_stage")
names(dd)[15]<-paste("m_stage")


dd<-dd %>% mutate(id = seq(1:nrow(dd)))
dd$id<-as.factor(dd$id)
dd$t_stage3<-as.factor(ifelse(dd$t_stage<4,1,
                              ifelse(dd$t_stage>6,3,2)))

dd$n_stage2<-as.factor(ifelse(dd$n_stage<2,1,2))
dd$m_stage2<-as.factor(ifelse(dd$m_stage<2,1,2))
```

#data inspection

```
str(dd)
```

```
## 'data.frame':    166 obs. of  23 variables:
##  $ patient_number: Factor w/ 165 levels "115847","128013",..: 165 164 163 162 161 160 159 158 157 156 ...
##  $ age           : int  71 78 68 61 48 65 68 62 75 53 ...
##  $ sex           : Factor w/ 2 levels "f","m": 2 1 2 1 2 2 2 1 2 2 ...
##  $ ht            : num  158 151 161 161 171 ...
##  $ wt            : num  65 65 70 80 72.4 ...
##  $ bmi           : num  26.2 28.4 27 30.9 24.9 ...
##  $ asa           : Factor w/ 4 levels "1","2","3","4": 2 2 2 2 1 2 2 2 2 2 ...
##  $ diagnosis     : Factor w/ 5 levels "colon","liver",..: 3 3 3 3 5 3 1 2 2 4 ...
##  $ pre_chemo     : Factor w/ 2 levels "0","1": 1 1 1 1 1 1 1 1 1 1 ...
##  $ pre_rt        : Factor w/ 2 levels "0","1": 1 1 1 1 1 1 1 1 1 1 ...
##  $ anesthetics   : Factor w/ 2 levels "inhalation","tiva": 1 1 1 1 2 1 2 1 1 1 ...
##  $ pathology     : Factor w/ 15 levels "a","Adenocarcinoma",..: 2 2 2 2 2 2 2 2 2 2 ...
##  $ t_stage       : int  2 8 8 7 8 5 7 7 7 8 ...
##  $ n_stage       : int  1 1 1 1 1 1 1 1 1 1 ...
##  $ m_stage       : int  1 1 1 1 1 1 1 1 1 1 ...
##  $ var_1         : num  0 0 0 0 0 0 0 0 0 0.03 ...
##  $ var_2         : num  0.096 0 0 0.055 0 0 0 0.047 0 0 ...
##  $ var_3         : num  0 0.033 1.169 0 0 ...
##  $ asahigh       : Factor w/ 2 levels "0","1": 1 1 1 1 1 1 1 1 1 1 ...
##  $ id            : Factor w/ 166 levels "1","2","3","4",..: 1 2 3 4 5 6 7 8 9 10 ...
##  $ t_stage3      : Factor w/ 3 levels "1","2","3": 1 3 3 3 3 2 3 3 3 3 ...
##  $ n_stage2      : Factor w/ 2 levels "1","2": 1 1 1 1 1 1 1 1 1 1 ...
##  $ m_stage2      : Factor w/ 2 levels "1","2": 1 1 1 1 1 1 1 1 1 1 ...
```

```
summary(dd)
```

```
##  patient_number      age        sex           ht              wt        
##  639376 :  2    Min.   :37.00   f: 45   Min.   :144.1   Min.   : 43.00  
##  115847 :  1    1st Qu.:61.25   m:121   1st Qu.:155.9   1st Qu.: 55.64  
##  128013 :  1    Median :68.00           Median :163.1   Median : 62.50  
##  156024 :  1    Mean   :66.17           Mean   :162.1   Mean   : 63.50  
##  164663 :  1    3rd Qu.:73.00           3rd Qu.:167.6   3rd Qu.: 70.00  
##  193431 :  1    Max.   :78.00           Max.   :180.8   Max.   :105.00  
##  (Other):159                                                            
##       bmi        asa        diagnosis  pre_chemo pre_rt      anesthetics 
##  Min.   :15.62   1:  8   colon   :89   0:160     0:160   inhalation:129  
##  1st Qu.:21.40   2:132   liver   :13   1:  6     1:  6   tiva      : 37  
##  Median :24.21   3: 24   lung    :39                                     
##  Mean   :24.15   4:  2   prostate:18                                     
##  3rd Qu.:26.48           renal   : 7                                     
##  Max.   :33.55                                                           
##                                                                          
##            pathology     t_stage         n_stage         m_stage     
##  a              :74   Min.   :1.000   Min.   :1.000   Min.   :1.000  
##  Adenocarcinoma :54   1st Qu.:3.000   1st Qu.:1.000   1st Qu.:1.000  
##  hcc            :10   Median :7.000   Median :1.000   Median :1.000  
##  scc            :10   Mean   :5.922   Mean   :1.633   Mean   :1.247  
##  ccc            : 5   3rd Qu.:8.000   3rd Qu.:1.000   3rd Qu.:1.000  
##  B-cell lymphoma: 2   Max.   :9.000   Max.   :7.000   Max.   :8.000  
##  (Other)        :11                                                  
##      var_1             var_2             var_3        asahigh       id     
##  Min.   :0.00000   Min.   : 0.0000   Min.   :0.0000   0:140   1      :  1  
##  1st Qu.:0.00000   1st Qu.: 0.0000   1st Qu.:0.0000   1: 26   2      :  1  
##  Median :0.09342   Median : 0.1332   Median :0.1294           3      :  1  
##  Mean   :0.30306   Mean   : 0.5732   Mean   :0.4243           4      :  1  
##  3rd Qu.:0.38450   3rd Qu.: 0.3686   3rd Qu.:0.4088           5      :  1  
##  Max.   :7.60000   Max.   :15.2000   Max.   :6.3000           6      :  1  
##                                                               (Other):160  
##  t_stage3 n_stage2 m_stage2
##  1:44     1:131    1:155   
##  2:32     2: 35    2: 11   
##  3:90                      
##                            
##                            
##                            
##
```

```
mytable(dd,method=3)
```

```
## 
##                          Descriptive Statistics                        
## ------------------------------------------------------------------------ 
##                                                   N         Total       
## ------------------------------------------------------------------------ 
##  patient_number                                 166  unique values  165 
##  age                                            166   68.0  [61.0;73.0] 
##  sex                                            166                     
##    - f                                                      45  (27.1%) 
##    - m                                                     121  (72.9%) 
##  ht                                             166        162.1 ± 8.1 
##  wt                                             166   62.5  [55.6;70.0] 
##  bmi                                            166         24.2 ± 3.5 
##  asa                                            166                     
##    - 1                                                        8  (4.8%) 
##    - 2                                                     132  (79.5%) 
##    - 3                                                      24  (14.5%) 
##    - 4                                                        2  (1.2%) 
##  diagnosis                                      166                     
##    - colon                                                  89  (53.6%) 
##    - liver                                                   13  (7.8%) 
##    - lung                                                   39  (23.5%) 
##    - prostate                                               18  (10.8%) 
##    - renal                                                    7  (4.2%) 
##  pre_chemo                                      166                     
##    - 0                                                     160  (96.4%) 
##    - 1                                                        6  (3.6%) 
##  pre_rt                                         166                     
##    - 0                                                     160  (96.4%) 
##    - 1                                                        6  (3.6%) 
##  anesthetics                                    166                     
##    - inhalation                                            129  (77.7%) 
##    - tiva                                                   37  (22.3%) 
##  pathology                                      166                     
##    - a                                                      74  (44.6%) 
##    - Adenocarcinoma                                         54  (32.5%) 
##    - adenosquamous carcinoma                                  1  (0.6%) 
##    - B-cell lymphoma                                          2  (1.2%) 
##    - ccc                                                      5  (3.0%) 
##    - cholangiocarcinoma                                       2  (1.2%) 
##    - chromophobe RCC                                          1  (0.6%) 
##    - hcc                                                     10  (6.0%) 
##    - Infiltrating urothelial carcinoma                        1  (0.6%) 
##    - Large cell neuroendocrine carcinoma                      1  (0.6%) 
##    - malignant solitary fibrous tumor                         1  (0.6%) 
##    - Metastatic adenocarcinoma                                1  (0.6%) 
##    - scc                                                     10  (6.0%) 
##    - small cell carcinoma                                     1  (0.6%) 
##    - tubular adenoma with high grade dysplasia                2  (1.2%) 
##  t_stage                                        166      7.0  [3.0;8.0] 
##  n_stage                                        166      1.0  [1.0;1.0] 
##  m_stage                                        166                     
##    - 1                                                     155  (93.4%) 
##    - 4                                                        9  (5.4%) 
##    - 8                                                        2  (1.2%) 
##  var_1                                          166      0.1  [0.0;0.4] 
##  var_2                                          166      0.1  [0.0;0.4] 
##  var_3                                          166      0.1  [0.0;0.4] 
##  asahigh                                        166                     
##    - 0                                                     140  (84.3%) 
##    - 1                                                      26  (15.7%) 
##  id                                             166  unique values  166 
##  t_stage3                                       166                     
##    - 1                                                      44  (26.5%) 
##    - 2                                                      32  (19.3%) 
##    - 3                                                      90  (54.2%) 
##  n_stage2                                       166                     
##    - 1                                                     131  (78.9%) 
##    - 2                                                      35  (21.1%) 
##  m_stage2                                       166                     
##    - 1                                                     155  (93.4%) 
##    - 2                                                       11  (6.6%) 
## ------------------------------------------------------------------------
```

##transform to long form

```
dd_long<-gather(dd,time,value,var_1:var_3,factor_key = T)
str(dd_long)
```

```
## 'data.frame':    498 obs. of  22 variables:
##  $ patient_number: Factor w/ 165 levels "115847","128013",..: 165 164 163 162 161 160 159 158 157 156 ...
##  $ age           : int  71 78 68 61 48 65 68 62 75 53 ...
##  $ sex           : Factor w/ 2 levels "f","m": 2 1 2 1 2 2 2 1 2 2 ...
##  $ ht            : num  158 151 161 161 171 ...
##  $ wt            : num  65 65 70 80 72.4 ...
##  $ bmi           : num  26.2 28.4 27 30.9 24.9 ...
##  $ asa           : Factor w/ 4 levels "1","2","3","4": 2 2 2 2 1 2 2 2 2 2 ...
##  $ diagnosis     : Factor w/ 5 levels "colon","liver",..: 3 3 3 3 5 3 1 2 2 4 ...
##  $ pre_chemo     : Factor w/ 2 levels "0","1": 1 1 1 1 1 1 1 1 1 1 ...
##  $ pre_rt        : Factor w/ 2 levels "0","1": 1 1 1 1 1 1 1 1 1 1 ...
##  $ anesthetics   : Factor w/ 2 levels "inhalation","tiva": 1 1 1 1 2 1 2 1 1 1 ...
##  $ pathology     : Factor w/ 15 levels "a","Adenocarcinoma",..: 2 2 2 2 2 2 2 2 2 2 ...
##  $ t_stage       : int  2 8 8 7 8 5 7 7 7 8 ...
##  $ n_stage       : int  1 1 1 1 1 1 1 1 1 1 ...
##  $ m_stage       : int  1 1 1 1 1 1 1 1 1 1 ...
##  $ asahigh       : Factor w/ 2 levels "0","1": 1 1 1 1 1 1 1 1 1 1 ...
##  $ id            : Factor w/ 166 levels "1","2","3","4",..: 1 2 3 4 5 6 7 8 9 10 ...
##  $ t_stage3      : Factor w/ 3 levels "1","2","3": 1 3 3 3 3 2 3 3 3 3 ...
##  $ n_stage2      : Factor w/ 2 levels "1","2": 1 1 1 1 1 1 1 1 1 1 ...
##  $ m_stage2      : Factor w/ 2 levels "1","2": 1 1 1 1 1 1 1 1 1 1 ...
##  $ time          : Factor w/ 3 levels "var_1","var_2",..: 1 1 1 1 1 1 1 1 1 1 ...
##  $ value         : num  0 0 0 0 0 0 0 0 0 0.03 ...
```

```
summary(dd)
```

```
##  patient_number      age        sex           ht              wt        
##  639376 :  2    Min.   :37.00   f: 45   Min.   :144.1   Min.   : 43.00  
##  115847 :  1    1st Qu.:61.25   m:121   1st Qu.:155.9   1st Qu.: 55.64  
##  128013 :  1    Median :68.00           Median :163.1   Median : 62.50  
##  156024 :  1    Mean   :66.17           Mean   :162.1   Mean   : 63.50  
##  164663 :  1    3rd Qu.:73.00           3rd Qu.:167.6   3rd Qu.: 70.00  
##  193431 :  1    Max.   :78.00           Max.   :180.8   Max.   :105.00  
##  (Other):159                                                            
##       bmi        asa        diagnosis  pre_chemo pre_rt      anesthetics 
##  Min.   :15.62   1:  8   colon   :89   0:160     0:160   inhalation:129  
##  1st Qu.:21.40   2:132   liver   :13   1:  6     1:  6   tiva      : 37  
##  Median :24.21   3: 24   lung    :39                                     
##  Mean   :24.15   4:  2   prostate:18                                     
##  3rd Qu.:26.48           renal   : 7                                     
##  Max.   :33.55                                                           
##                                                                          
##            pathology     t_stage         n_stage         m_stage     
##  a              :74   Min.   :1.000   Min.   :1.000   Min.   :1.000  
##  Adenocarcinoma :54   1st Qu.:3.000   1st Qu.:1.000   1st Qu.:1.000  
##  hcc            :10   Median :7.000   Median :1.000   Median :1.000  
##  scc            :10   Mean   :5.922   Mean   :1.633   Mean   :1.247  
##  ccc            : 5   3rd Qu.:8.000   3rd Qu.:1.000   3rd Qu.:1.000  
##  B-cell lymphoma: 2   Max.   :9.000   Max.   :7.000   Max.   :8.000  
##  (Other)        :11                                                  
##      var_1             var_2             var_3        asahigh       id     
##  Min.   :0.00000   Min.   : 0.0000   Min.   :0.0000   0:140   1      :  1  
##  1st Qu.:0.00000   1st Qu.: 0.0000   1st Qu.:0.0000   1: 26   2      :  1  
##  Median :0.09342   Median : 0.1332   Median :0.1294           3      :  1  
##  Mean   :0.30306   Mean   : 0.5732   Mean   :0.4243           4      :  1  
##  3rd Qu.:0.38450   3rd Qu.: 0.3686   3rd Qu.:0.4088           5      :  1  
##  Max.   :7.60000   Max.   :15.2000   Max.   :6.3000           6      :  1  
##                                                               (Other):160  
##  t_stage3 n_stage2 m_stage2
##  1:44     1:131    1:155   
##  2:32     2: 35    2: 11   
##  3:90                      
##                            
##                            
##                            
##
```

#plot

```
#tiva vs inhalation (original scale)
ggplot(dd_long,aes(x=time,y=value,col=anesthetics))+
  geom_boxplot()
```

```
#tiva vs inhalation (log transformed)
ggplot(dd_long,aes(x=time,y=value,col=anesthetics))+
  scale_y_log10(breaks = trans_breaks("log10", function(x) 10^x),
                labels = trans_format("log10", math_format(10^.x)))+
  geom_boxplot()
```

```
ggplot(dd_long,aes(x=time,y=value,group=anesthetics,col=anesthetics))+
  geom_smooth()+
  scale_y_log10(breaks = trans_breaks("log10", function(x) 10^x),
                labels = trans_format("log10", math_format(10^.x)))+
  stat_summary(aes(group =anesthetics),geom = "point",fun.y = mean,
               shape = 17, size = 3)
```

```
## `geom_smooth()` using method = 'loess' and formula 'y ~ x'
```

```
#by diagnosis (original scale)
ggplot(dd_long,aes(x=time,y=value,col=diagnosis))+
  geom_boxplot(add="jitter")
```

```
#by diagnosis (log transformed)
ggplot(dd_long,aes(x=time,y=value,col=diagnosis))+
  scale_y_log10(breaks = trans_breaks("log10", function(x) 10^x),
                labels = trans_format("log10", math_format(10^.x)))+
  geom_boxplot(add="jitter")
```

```
ggplot(dd_long,aes(x=time,y=value,group=diagnosis,col=diagnosis))+
  geom_smooth()+
  scale_y_log10(breaks = trans_breaks("log10", function(x) 10^x),
                labels = trans_format("log10", math_format(10^.x)))+
  stat_summary(aes(group =diagnosis),geom = "point",fun.y = mean,
               shape = 17, size = 3)
```

```
## `geom_smooth()` using method = 'loess' and formula 'y ~ x'
```

#mytable

```
mytable(anesthetics~var_1+var_2+var_3,dd,method=3)
```

```
## 
##    Descriptive Statistics by 'anesthetics'   
## ______________________________________________ 
##           inhalation          tiva         p  
##            (N=129)           (N=37)     
## ---------------------------------------------- 
##  var_1  0.1 [ 0.0; 0.4]  0.0 [ 0.0; 0.2] 0.089
##  var_2  0.2 [ 0.0; 0.4]  0.1 [ 0.0; 0.3] 0.250
##  var_3  0.1 [ 0.0; 0.5]  0.1 [ 0.0; 0.3] 0.290
## ----------------------------------------------
```

```
mytable(diagnosis~var_1+var_2+var_3,dd,method=3)
```

```
## 
##                               Descriptive Statistics by 'diagnosis'                             
## _________________________________________________________________________________________________ 
##             colon            liver             lung           prostate          renal         p  
##             (N=89)           (N=13)           (N=39)           (N=18)           (N=7)      
## ------------------------------------------------------------------------------------------------- 
##  var_1  0.2 [ 0.0; 0.4]  0.1 [ 0.0; 0.2]  0.0 [ 0.0; 0.3]  0.0 [ 0.0; 0.2]  0.0 [ 0.0; 0.2] 0.049
##  var_2  0.2 [ 0.0; 0.4]  0.0 [ 0.0; 0.4]  0.1 [ 0.0; 0.3]  0.1 [ 0.0; 0.3]  0.0 [ 0.0; 0.1] 0.075
##  var_3  0.2 [ 0.0; 0.5]  0.0 [ 0.0; 0.2]  0.1 [ 0.0; 0.4]  0.0 [ 0.0; 0.5]  0.0 [ 0.0; 0.1] 0.113
## -------------------------------------------------------------------------------------------------
```

#time effect

```
fit<-aov(value~time+Error(id/time),data=dd_long) 
summary(fit)
```

```
## 
## Error: id
##            Df Sum Sq Mean Sq F value Pr(>F)
## Residuals 165  480.6   2.913               
## 
## Error: id:time
##            Df Sum Sq Mean Sq F value Pr(>F)  
## time        2   6.08  3.0391   3.609 0.0282 *
## Residuals 330 277.93  0.8422                 
## ---
## Signif. codes:  0 '***' 0.001 '**' 0.01 '*' 0.05 '.' 0.1 ' ' 1
```

```
fit<-lmer(value~time+(1|id),data=dd_long)
anova(fit)
```

```
## Type III Analysis of Variance Table with Satterthwaite's method
##      Sum Sq Mean Sq NumDF DenDF F value  Pr(>F)  
## time 6.0782  3.0391     2   330  3.6086 0.02817 *
## ---
## Signif. codes:  0 '***' 0.001 '**' 0.01 '*' 0.05 '.' 0.1 ' ' 1
```

#time \* anesthetics interaction model

```
fit<-lmer(value~time*anesthetics+(1|id),data=dd_long)
anova(fit)
```

```
## Type III Analysis of Variance Table with Satterthwaite's method
##                  Sum Sq Mean Sq NumDF DenDF F value   Pr(>F)   
## time             8.7896  4.3948     2   328  5.2378 0.005765 **
## anesthetics      0.0376  0.0376     1   164  0.0448 0.832702   
## time:anesthetics 2.7164  1.3582     2   328  1.6187 0.199734   
## ---
## Signif. codes:  0 '***' 0.001 '**' 0.01 '*' 0.05 '.' 0.1 ' ' 1
```

```
fit<-lmer(log1p(value)~time*anesthetics+(1|id),data=dd_long)
anova(fit)
```

```
## Type III Analysis of Variance Table with Satterthwaite's method
##                   Sum Sq Mean Sq NumDF DenDF F value  Pr(>F)  
## time             0.68117 0.34058     2   328  4.1073 0.01731 *
## anesthetics      0.02443 0.02443     1   164  0.2946 0.58801  
## time:anesthetics 0.19594 0.09797     2   328  1.1815 0.30813  
## ---
## Signif. codes:  0 '***' 0.001 '**' 0.01 '*' 0.05 '.' 0.1 ' ' 1
```

#time \* diagnosis interaction model

```
fit<-lmer(value~time*diagnosis+(1|id),data=dd_long)
anova(fit)
```

```
## Type III Analysis of Variance Table with Satterthwaite's method
##                Sum Sq Mean Sq NumDF DenDF F value Pr(>F)
## time           2.7625 1.38123     2   322  1.6307 0.1974
## diagnosis      2.5193 0.62983     4   161  0.7436 0.5636
## time:diagnosis 5.1783 0.64728     8   322  0.7642 0.6347
```

```
fit<-lmer(log1p(value)~time*diagnosis+(1|id),data=dd_long)
anova(fit)
```

```
## Type III Analysis of Variance Table with Satterthwaite's method
##                 Sum Sq Mean Sq NumDF DenDF F value Pr(>F)
## time           0.31427 0.15713     2   322  1.9128 0.1493
## diagnosis      0.44868 0.11217     4   161  1.3654 0.2483
## time:diagnosis 0.94138 0.11767     8   322  1.4324 0.1819
```

#time \* stage interaction models

```
fit<-lmer(log1p(value)~time*t_stage+(1|id),data=dd_long)
anova(fit)
```

```
## Type III Analysis of Variance Table with Satterthwaite's method
##                Sum Sq  Mean Sq NumDF DenDF F value Pr(>F)
## time         0.001628 0.000814     2   328  0.0098 0.9903
## t_stage      0.017790 0.017790     1   164  0.2135 0.6446
## time:t_stage 0.066652 0.033326     2   328  0.4000 0.6706
```

```
fit<-lmer(value~time*n_stage+(1|id),data=dd_long)
anova(fit)
```

```
## Type III Analysis of Variance Table with Satterthwaite's method
##               Sum Sq Mean Sq NumDF DenDF F value Pr(>F)
## time         3.05088 1.52544     2   328  1.8012 0.1667
## n_stage      0.00219 0.00219     1   164  0.0026 0.9595
## time:n_stage 0.13496 0.06748     2   328  0.0797 0.9234
```

```
fit<-lmer(value~time*m_stage+(1|id),data=dd_long)
anova(fit)
```

```
## Type III Analysis of Variance Table with Satterthwaite's method
##              Sum Sq Mean Sq NumDF DenDF F value  Pr(>F)  
## time         1.5201 0.76006     2   328  0.9011 0.40714  
## m_stage      2.8726 2.87262     1   164  3.4056 0.06678 .
## time:m_stage 1.2568 0.62841     2   328  0.7450 0.47554  
## ---
## Signif. codes:  0 '***' 0.001 '**' 0.01 '*' 0.05 '.' 0.1 ' ' 1
```

##plot2

```
Sum = groupwiseMean(value ~ anesthetics + time,
                    data   = dd_long,
                    conf   = 0.95,
                    digits = 3,
                    traditional = FALSE,
                    percentile  = TRUE)

pd = position_dodge(.2)

ggplot(Sum, aes(x =    time,
                y =    Mean,
                color = anesthetics)) +
  geom_errorbar(aes(ymin=Percentile.lower,
                    ymax=Percentile.upper),
                width=.2, size=0.7, position=pd) +
  geom_point(shape=15, size=4, position=pd) +
  theme_bw() +
  scale_y_log10(breaks = trans_breaks("log10", function(x) 10^x),
                labels = trans_format("log10", math_format(10^.x)))+
  
  theme(axis.title = element_text(face = "bold")) +
  ylab("value")
```

```
##plot2 (by dx)
Sum = groupwiseMean(value ~ diagnosis + time,
                    data   = dd_long,
                    conf   = 0.95,
                    digits = 3,
                    traditional = FALSE,
                    percentile  = TRUE)

pd = position_dodge(.3)

ggplot(Sum, aes(x =    time,
                y =    Mean,
                color = diagnosis)) +
  geom_errorbar(aes(ymin=Percentile.lower,
                    ymax=Percentile.upper),
                width=.2, size=0.7, position=pd) +
  geom_point(shape=15, size=4, position=pd) +
  scale_y_log10(breaks = trans_breaks("log10", function(x) 10^x),
                labels = trans_format("log10", math_format(10^.x)))+
  theme_bw() +
  theme(axis.title = element_text(face = "bold")) +
  ylab("value")
```

##statistician’s analysis

```
### mvn 으로는 그룹 별로 정규성을 보기 힘듦
mvn(dd_long[,c(2,4,5,6,13,14,15,22)], mvnTest = "mardia")
```

```
## $multivariateNormality
##              Test        Statistic p value Result
## 1 Mardia Skewness 7853.48558897395       0     NO
## 2 Mardia Kurtosis 92.7063692987946       0     NO
## 3             MVN             <NA>    <NA>     NO
## 
## $univariateNormality
##           Test  Variable Statistic   p value Normality
## 1 Shapiro-Wilk    age       0.9322  <0.001      NO    
## 2 Shapiro-Wilk    ht        0.9890   8e-04      NO    
## 3 Shapiro-Wilk    wt        0.9755  <0.001      NO    
## 4 Shapiro-Wilk    bmi       0.9942  0.0537      YES   
## 5 Shapiro-Wilk  t_stage     0.8322  <0.001      NO    
## 6 Shapiro-Wilk  n_stage     0.5201  <0.001      NO    
## 7 Shapiro-Wilk  m_stage     0.2574  <0.001      NO    
## 8 Shapiro-Wilk   value      0.3289  <0.001      NO    
## 
## $Descriptives
##           n        Mean   Std.Dev      Median    Min    Max   25th     75th
## age     498  66.1686747  8.748421  68.0000000  37.00  78.00  61.00  73.0000
## ht      498 162.1084337  8.097361 163.1000000 144.10 180.80 155.90 167.6000
## wt      498  63.4978916 10.500136  62.5000000  43.00 105.00  55.60  70.0000
## bmi     498  24.1520482  3.443723  24.2100000  15.62  33.55  21.36  26.4900
## t_stage 498   5.9216867  2.541386   7.0000000   1.00   9.00   3.00   8.0000
## n_stage 498   1.6325301  1.391085   1.0000000   1.00   7.00   1.00   1.0000
## m_stage 498   1.2469880  1.009632   1.0000000   1.00   8.00   1.00   1.0000
## value   498   0.4335189  1.240319   0.1192188   0.00  15.20   0.00   0.3925
##                Skew   Kurtosis
## age     -0.90776143  0.5530866
## ht      -0.06049273 -0.5446959
## wt       0.59576845  0.5989063
## bmi      0.09961015 -0.2909941
## t_stage -0.51364723 -1.2870751
## n_stage  2.20350521  3.8588199
## m_stage  4.69067462 24.1139802
## value    7.45142501 70.2581730
```

```
shapiro.test(dd_long$value)
```

```
## 
##  Shapiro-Wilk normality test
## 
## data:  dd_long$value
## W = 0.3289, p-value < 2.2e-16
```

```
boxplot(dd_long$value)
```

```
boxplot(dd_long$value~dd_long$time)
```

```
# transformed 'value'
shapiro.test(log10(dd_long$value+1))
```

```
## 
##  Shapiro-Wilk normality test
## 
## data:  log10(dd_long$value + 1)
## W = 0.64812, p-value < 2.2e-16
```

```
boxplot(log10(dd_long$value+1)~dd_long$time)
```

```
dd_long$val.trans = log10(dd_long$value+1)

### 그룹 별로 각각 정규성 
shapiro.test(dd_long$val.trans[which(dd_long$time=="var_1")])
```

```
## 
##  Shapiro-Wilk normality test
## 
## data:  dd_long$val.trans[which(dd_long$time == "var_1")]
## W = 0.67468, p-value < 2.2e-16
```

```
shapiro.test(dd_long$val.trans[which(dd_long$time=="var_2")])
```

```
## 
##  Shapiro-Wilk normality test
## 
## data:  dd_long$val.trans[which(dd_long$time == "var_2")]
## W = 0.60528, p-value < 2.2e-16
```

```
shapiro.test(dd_long$val.trans[which(dd_long$time=="var_3")])
```

```
## 
##  Shapiro-Wilk normality test
## 
## data:  dd_long$val.trans[which(dd_long$time == "var_3")]
## W = 0.70705, p-value < 2.2e-16
```

```
### kruskal test  
kruskal.test(val.trans~time, data=dd_long)
```

```
## 
##  Kruskal-Wallis rank sum test
## 
## data:  val.trans by time
## Kruskal-Wallis chi-squared = 1.5392, df = 2, p-value = 0.4632
```

```
kruskal.test(val.trans~diagnosis, data=dd_long)
```

```
## 
##  Kruskal-Wallis rank sum test
## 
## data:  val.trans by diagnosis
## Kruskal-Wallis chi-squared = 21.21, df = 4, p-value = 0.0002878
```

```
kruskal.test(val.trans~pathology, data=dd_long)
```

```
## 
##  Kruskal-Wallis rank sum test
## 
## data:  val.trans by pathology
## Kruskal-Wallis chi-squared = 63.798, df = 14, p-value = 2.505e-08
```

```
# by time 
kruskal.test(val.trans~diagnosis, data=dd_long[dd_long$time=="var_1",])
```

```
## 
##  Kruskal-Wallis rank sum test
## 
## data:  val.trans by diagnosis
## Kruskal-Wallis chi-squared = 9.5438, df = 4, p-value = 0.04886
```

```
kruskal.test(val.trans~diagnosis, data=dd_long[dd_long$time=="var_2",])
```

```
## 
##  Kruskal-Wallis rank sum test
## 
## data:  val.trans by diagnosis
## Kruskal-Wallis chi-squared = 8.4827, df = 4, p-value = 0.07541
```

```
kruskal.test(val.trans~diagnosis, data=dd_long[dd_long$time=="var_3",])
```

```
## 
##  Kruskal-Wallis rank sum test
## 
## data:  val.trans by diagnosis
## Kruskal-Wallis chi-squared = 7.4688, df = 4, p-value = 0.1131
```

```
kruskal.test(val.trans~pathology, data=dd_long[dd_long$time=="var_1",])
```

```
## 
##  Kruskal-Wallis rank sum test
## 
## data:  val.trans by pathology
## Kruskal-Wallis chi-squared = 32.594, df = 14, p-value = 0.003295
```

```
kruskal.test(val.trans~pathology, data=dd_long[dd_long$time=="var_2",])
```

```
## 
##  Kruskal-Wallis rank sum test
## 
## data:  val.trans by pathology
## Kruskal-Wallis chi-squared = 22.051, df = 14, p-value = 0.07758
```

```
kruskal.test(val.trans~pathology, data=dd_long[dd_long$time=="var_3",])
```

```
## 
##  Kruskal-Wallis rank sum test
## 
## data:  val.trans by pathology
## Kruskal-Wallis chi-squared = 22.753, df = 14, p-value = 0.06441
```

```
kruskal.test(val.trans ~ anesthetics, data = dd_long[dd_long$time=="var_1",])
```

```
## 
##  Kruskal-Wallis rank sum test
## 
## data:  val.trans by anesthetics
## Kruskal-Wallis chi-squared = 2.8942, df = 1, p-value = 0.0889
```

```
kruskal.test(val.trans ~ anesthetics, data = dd_long[dd_long$time=="var_2",])
```

```
## 
##  Kruskal-Wallis rank sum test
## 
## data:  val.trans by anesthetics
## Kruskal-Wallis chi-squared = 1.329, df = 1, p-value = 0.249
```

```
kruskal.test(val.trans ~ anesthetics, data = dd_long[dd_long$time=="var_3",])
```

```
## 
##  Kruskal-Wallis rank sum test
## 
## data:  val.trans by anesthetics
## Kruskal-Wallis chi-squared = 1.1227, df = 1, p-value = 0.2893
```

```
#id vs time
friedman03 = aggregate(x = dd_long$value,
                       by = list(id = dd_long$id, time = dd_long$time),
                       FUN = median)
friedman.test(x ~ time | id, data = friedman03)
```

```
## 
##  Friedman rank sum test
## 
## data:  x and time and id
## Friedman chi-squared = 5.2044, df = 2, p-value = 0.07411
```

```
#anesthetics vs time
friedman04 = aggregate(x = dd_long$value,
                       by = list(anesthetics = dd_long$anesthetics, time = dd_long$time),
                       FUN = median)
friedman.test(x ~ time | anesthetics, data = friedman04)
```

```
## 
##  Friedman rank sum test
## 
## data:  x and time and anesthetics
## Friedman chi-squared = 3, df = 2, p-value = 0.2231
```

```
#diagnosis vs time
friedman05 = aggregate(x = dd_long$value,
                       by = list(diagnosis = dd_long$diagnosis, time = dd_long$time),
                       FUN = median)
friedman.test(x ~ time | diagnosis, data = friedman05)
```

```
## 
##  Friedman rank sum test
## 
## data:  x and time and diagnosis
## Friedman chi-squared = 2, df = 2, p-value = 0.3679
```
